# Plant stem cell research is uncovering the secrets of longevity and persistent growth

**DOI:** 10.1111/tpj.15184

**Published:** 2021-03-25

**Authors:** Masaaki Umeda, Momoko Ikeuchi, Masaki Ishikawa, Toshiro Ito, Ryuichi Nishihama, Junko Kyozuka, Keiko U. Torii, Akiko Satake, Gohta Goshima, Hitoshi Sakakibara

**Affiliations:** ^1^ Graduate School of Science and Technology Nara Institute of Science and Technology Ikoma 630‐0192 Japan; ^2^ Department of Biology Faculty of Science Niigata University Niigata 950‐2181 Japan; ^3^ National Institute for Basic Biology Okazaki 444‐8585 Japan; ^4^ Department of Basic Biology The Graduate University for Advanced Studies (SOKENDAI) Okazaki 444‐8585 Japan; ^5^ Graduate School of Biostudies Kyoto University Kyoto 606‐8502 Japan; ^6^ Graduate School of Life Sciences Tohoku University Sendai 980‐8577 Japan; ^7^ Howard Hughes Medical Institute and Department of Molecular Biosciences The University of Texas at Austin Austin TX 78712 USA; ^8^ Institute of Transformative Biomolecules (WPI‐ITbM) Nagoya University Nagoya 464‐8601 Japan; ^9^ Department of Biology Faculty of Science Kyushu University Fukuoka 819‐0395 Japan; ^10^ Division of Biological Science Graduate School of Science Nagoya University Nagoya 464‐8602 Japan; ^11^ Sugashima Marine Biological Laboratory Graduate School of Science Nagoya University Toba 517‐0004 Japan; ^12^ Graduate School of Bioagricultural Sciences Nagoya University Nagoya 464‐8601 Japan

**Keywords:** stem cell, pluripotency, reprogramming, meristem, asymmetric cell division, genome stability

## Abstract

Plant stem cells have several extraordinary features: they are generated *de novo* during development and regeneration, maintain their pluripotency, and produce another stem cell niche in an orderly manner. This enables plants to survive for an extended period and to continuously make new organs, representing a clear difference in their developmental program from animals. To uncover regulatory principles governing plant stem cell characteristics, our research project ‘Principles of pluripotent stem cells underlying plant vitality’ was launched in 2017, supported by a Grant‐in‐Aid for Scientific Research on Innovative Areas from the Japanese government. Through a collaboration involving 28 research groups, we aim to identify key factors that trigger epigenetic reprogramming and global changes in gene networks, and thereby contribute to stem cell generation. Pluripotent stem cells in the shoot apical meristem are controlled by cytokinin and auxin, which also play a crucial role in terminating stem cell activity in the floral meristem; therefore, we are focusing on biosynthesis, metabolism, transport, perception, and signaling of these hormones. Besides, we are uncovering the mechanisms of asymmetric cell division and of stem cell death and replenishment under DNA stress, which will illuminate plant‐specific features in preserving stemness. Our technology support groups expand single‐cell omics to describe stem cell behavior in a spatiotemporal context, and provide correlative light and electron microscopic technology to enable live imaging of cell and subcellular dynamics at high spatiotemporal resolution. In this perspective, we discuss future directions of our ongoing projects and related research fields.

## Significance Statement

Plant stem cells are distinct from animal tissue stem cells in that they maintain pluripotency throughout life. Here we highlight achievements and future perspectives of our projects on stem cell research, including reprogramming, hormonal and epigenetic regulation of stemness, meristem determinacy, and the control of asymmetric division and genome integrity in plant stem cells.

## INTRODUCTION

Plants have remarkable longevity: some trees can live for up to thousands of years. Many plant species age rapidly after flowering, while without flowering they stay alive for a long time; for instance, perennial rice (*Oryza sativa*) plants can be multiplied by separating a parent plant before ear emergence, and if the gene encoding the flowering hormone ‘florigen’ is suppressed, individual plants can survive for many years (Komiya *et al.*, 2008). In addition, plants have the ability to continuously generate organs throughout life under fluctuating environments. These features enable plants to flourish all over the earth, thereby contributing to environmental preservation, the human food supply, and biomass production.

**Figure 1:**
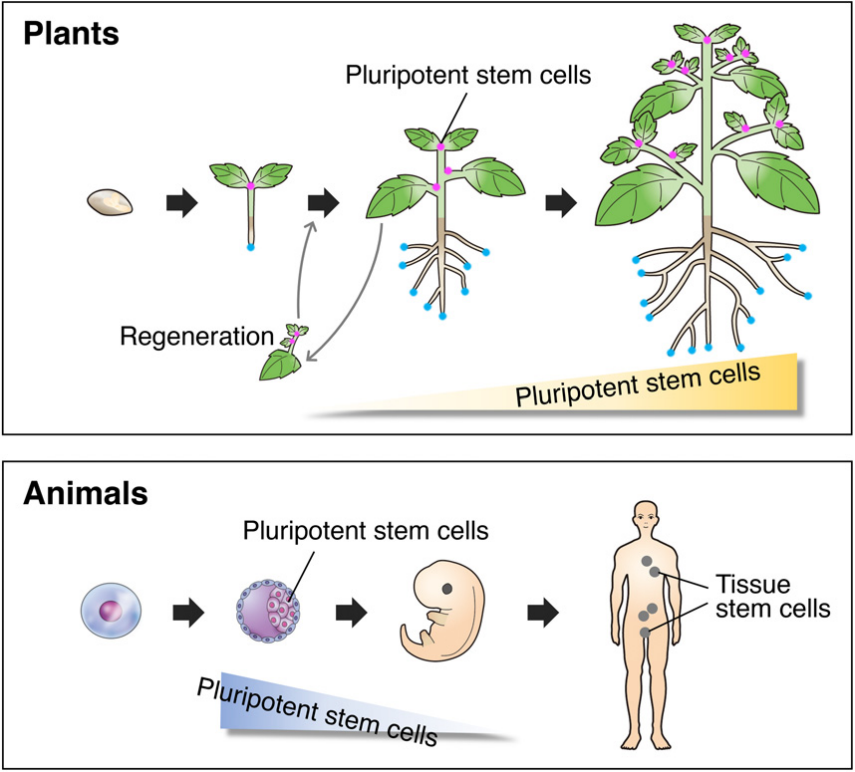
Stem cells in plants and animals. Stem cells in the apical and axillary meristems in shoots maintain pluripotency, and their population continuously increases in number during development (pink). Root stem cells are unipotent, but different types are cooperatively involved in root development (blue). In animals, pluripotent stem cells disappear soon after early embryogenesis, and, in the adult body, tissue (adult or somatic) stem cells differentiate into specific cell types and maintain tissue homeostasis.

The source of plant longevity and persistent growth is stem cells, which remain pluripotent and are preserved in tissues throughout life. In animals, pluripotent stem cells disappear soon after early embryogenesis, and, in the adult body, tissue homeostasis is maintained by multipotent stem cells, called tissue stem cells, that are capable of differentiating only into specific cell types (Figure 1). Consequently, post‐embryonic organ formation ceases at an early point during animal development. In contrast, plant pluripotent stem cells continuously proliferate to generate aboveground tissues, supporting sustained growth (Figure 1). Although each root stem cell is unipotent, root tip excision gives rise to regeneration of the stem cell niche above the excision point, implying a high reprogramming potential of root cells (Birnbaum, 2016). However, despite many genetic investigations over the last 30 years, our knowledge of the intrinsic properties of plant stem cells is limited, and their characteristics have been studied principally at the meristem level rather than the cellular level. As a result, how stem cell populations are augmented *in vivo* and how pluripotent stem cells are maintained over a prolonged period still remain fundamental questions in biology.

To address these questions, we launched a project focusing on plant stem cells in 2017, entitled ‘Principles of pluripotent stem cells underlying plant vitality’, which is supported by a Grant‐in‐Aid for Scientific Research on Innovative Areas from the Ministry of Education, Culture, Sports, Science and Technology, Japan (http://www.plant‐stem‐cells.jp/en/). Twenty‐eight researchers from different fields are studying plant stem cell proliferation and maintenance using *Arabidopsis thaliana*, *O. sativa*, *Lotus japonicus*, *Marchantia polymorpha*, *Physcomitrium* (*Physcomitrella*) *patens*, trees such as *Lithocarpus edulis* and *Betula platyphylla*, and more. Comparative analyses of different stem cell types in diverse plant species enable us to obtain key information about pluripotency and stemness. Several animal researchers also participate in the project and provide clues to compare the characteristic features of plant and animal stem cells, highlighting plant‐specific mechanisms underlying the maintenance of genome integrity. Two independent groups apply new technologies such as single‐cell analysis and correlative light and electron microscopy to draw a complete picture of stem cell behavior in a spatiotemporal context. In this article, we introduce some of our ongoing studies that will uncover the prominent features of plant stem cells and discuss recent findings and future perspectives in the research fields of reprogramming, hormonal and epigenetic regulation of stemness, meristem determinacy, asymmetric cell division, and genome stability.

## FORMATION OF STEM CELLS

Plant stem cells arise during embryogenesis and are preserved throughout life. Stem cells can also be generated *de novo*, as observed during lateral root formation. Besides stem cell formation in normal developmental programs, plants can easily generate stem cells during tissue regeneration (Figure 1). In most cases of tissue regeneration or wound healing in animals, tissue stem cells (e.g., neoblasts in planarians) are reactivated to form new organs or to replace lost parts of the body, and differentiated cells rarely acquire stem cell fate. In contrast, wounded plants are able to generate new stem cells with higher potency by reprogramming of differentiated cells with limited potency (Birnbaum and Sanchez Alvarado, 2008). At wound sites, flowering plants first activate cell division and form cell clumps called calli, which then generate stem cells for shoots or roots depending on their position within the plant body (Ichihashi *et al.*, 2020; Ikeuchi *et al.*, 2019). In tissue culture, these processes are often enhanced by exogenously supplied hormones, such as auxin and cytokinin.

**Figure 2:**
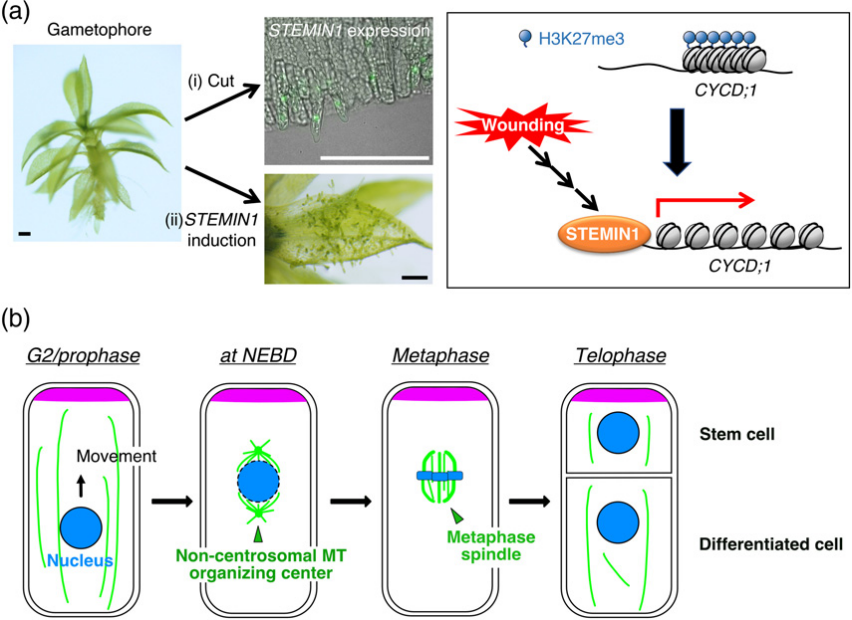
Stem cell formation and asymmetric division. (a) Formation of stem cells in *P. patens*. (i) When a leaf is excised from a gametophore, leaf cells facing the cut express *STEMIN1* (green) and subsequently convert into chloronema apical stem cells. (ii) When *STEMIN1* is induced in gametophores, leaf cells directly convert into chloronema apical stem cells without excision. Wounding induces STEMIN1, which then binds to the *CYCD;1* promoter and confers removal of H3K27me3 and concomitant induction of *CYCD;1* (right panel). Bars = 200 µm. (b) Asymmetric division of a stem cell. The dynamics of the microtubule (MT)‐based structures (green) and chromosomes (blue) are shown. Magenta represents asymmetrically localized polarity factors and fate determinants. In plants that do not possess centrosomes, non‐centrosomal microtubule organizing centers emerge during prophase and control metaphase spindle orientation. They are called the gametosome in moss, polar organizer (PO) in liverwort, and polar cap (or prospindle) in seed plants. The structures appear transiently and are no longer visible after nuclear envelope breakdown (NEBD).

Previous studies identified several APETALA2/ETHYLENE RESPONSE FACTOR (AP2/ERF) transcription factors as key regulators for regeneration that are quickly induced after wounding, such as WOUND INDUCED DEDIFFERENTIATION 1 (WIND1), ERF113/RELATED TO AP2 L (RAP2.6L), ERF109, and ERF115 in Arabidopsis (Che *et al.*, 2006; Iwase *et al.*, 2011; Zhang *et al.*, 2019; Zhou *et al.*, 2019). Some of them may act as ‘pioneer factors’ that engage silenced gene loci to render them accessible to epigenetic regulators for reprogramming in somatic cells (Iwafuchi‐Doi and Zaret, 2014), as Yamanaka factors do in differentiated mammalian cells to induce pluripotent stem cells. In *P. patens*, when a leaf is excised from a leafy shoot gametophore and cultivated on growth medium, cells facing the cut are reprogrammed into chloronema apical stem cells (Ishikawa *et al.*, 2011) (Figure 2a). Research from our project revealed that *STEM CELL‐INDUCING FACTOR 1* (*STEMIN1*), encoding an AP2/ERF transcription factor, is induced in cells undergoing reprogramming and that its ectopic expression in gametophores changes leaf cells into stem cells in the absence of wound signals (Ishikawa *et al.*, 2019) (Figure 2a). Removal of repressive trimethylation at lysine 27 of histone H3 (H3K27me3) was detected at genes directly targeted by STEMIN1, such as the D‐type cyclin gene *CYCD;1*, after STEMIN1 binding, suggesting a role for STEMIN1 as a pioneer factor (Ishikawa *et al.*, 2019) (Figure 2a). Other research demonstrated that wounding triggers histone H3 acetylation at a subset of wound‐induced genes including *RAP2.6L* (Rymen *et al.*, 2019). Interestingly, histone H3 located at early wound‐induced genes, such as *WIND1*, is acetylated prior to wounding, suggesting that these genes are on standby for a quick response to wounding.

Our project aims to answer the following three questions about *de novo* stem cell formation. (i) How does wounding activate key transcription factors? Recent studies demonstrated that in root regeneration, the defense‐related stress hormone jasmonate (JA) is elevated upon wounding and induces *ERF109* and *ERF115* expression (Zhang *et al.*, 2019; Zhou *et al.*, 2019). However, its role seems to be context‐dependent, as JA signaling inhibits callus formation after hypocotyl cutting (Ikeuchi *et al.*, 2017). We recently found DNA strand break‐induced reprogramming via *STEMIN1* induction in *P. patens* (Gu *et al.*, 2020), which provides a new perspective on the issue of DNA damage‐dependent regeneration. (ii) How are cell cycle progression and establishment of stem cell fate coupled? Reprogramming of differentiated cells into stem cells requires coordination between cell cycle progression and acquisition of new cell fate. Previous findings suggest that, in some cases, cell fate is altered without cell cycle activation; for instance, in *P. patens* leaves, cell fate changes independently of cell cycle progression (Ishikawa *et al.*, 2011), and shoot organogenesis in *Torenia fournieri* occurs directly from leaf explants without callus induction (Bridgen *et al.*, 1994). Therefore, even the nature or the sequence of molecular events for cell cycle activation and stem cell fate determination remain poorly understood thus far. We aim to answer these questions by comparing various experimental systems and taking single‐cell approaches. (iii) How is *de novo* stem cell formation regulated? In many angiosperm species, pericycle cells are known to have the remarkable capacity to give rise to stem cells, for example during lateral root formation or tissue culture‐based shoot meristem formation. Our project is now uncovering the molecular basis of this outstanding feature of pericycle cells to generate stem cells. Although many factors involved in organ development and stem cell maintenance have been shown to be associated with *de novo* stem cell formation, it remains unclear whether they have crucial roles in direct reprogramming of somatic cells into stem cells. The legume *L. japonicus* forms root nodules in response to infection by nitrogen‐fixing bacteria, which activates the cell cycle in the cortex by inducing the RWP‐RK transcription factor NODULE INCEPTION (NIN) and may trigger stem cell formation (Ferguson *et al.*, 2019; Ichihashi *et al.*, 2020). Gemma formation in *M. polymorpha* requires the MYB transcription factor GEMMA CUP‐ASSOCIATED MYB1 (GCAM1), overexpression of which generates a mass of undifferentiated cells (Yasui *et al.*, 2019). These transcription factors are likely to play a key role in *de novo* stem cell formation during development; thus, further studies will deepen our understanding of the mechanisms underlying the acquisition of stemness and shed light on the conservation and divergence of reprogramming machineries in land plants.

## EPIGENETIC CONTROL OF STEM CELLS

Recent cell type‐specific epigenome profiling has looked for characteristics of plant stem cells that are potentially embedded in chromatin status. Sijacic et al. (2018) collected nuclei from *CLAVATA3* (*CLV3*)‐expressing stem cells in the Arabidopsis shoot apical meristem (SAM) using the isolation of nuclei tagged in specific cell types (INTACT) nuclear capture method (Deal and Henikoff, 2010), and profiled chromatin accessibility by assay for transposase‐accessible chromatin sequencing (ATAC‐seq). Comparing the ATAC‐seq profile of the stem cells with that of differentiated mesophyll cells revealed that the two cell populations do not display a clear qualitative difference in most genomic loci, but rather preferential enrichment in each cell type (Sijacic *et al.*, 2018). Notably, ‘open’ chromatin regions enriched in differentiated cells are already open in stem cells. It is tempting to speculate that stem cells are already prepared for transcription factor binding signifying differentiation and that this feature is associated with the pluripotency of plant stem cells. Plants also safeguard the genome of stem cells against mobilization of transposable elements to ensure faithful inheritance of their genetic information. By transcriptome and DNA methylome profiling of stem cells within the SAM, Gutzat *et al*. (2020) revealed that transposable elements become increasingly methylated in the CHG context and transcriptionally silenced as Arabidopsis plants undergo the transition from vegetative to reproductive stage. Higo *et al*. (2020) observed a similar trend of increased methylation in the CHH context in the rice meristem, together suggesting that plants modulate the DNA methylation status of stem cells, thereby maintaining genome integrity, according to developmental stages.

Given that stem cells have some discernible characteristic features in their epigenome status, it is of great interest to illuminate epigenome reconfiguration during *de novo* establishment of stem cells from somatic cells or during cellular reprogramming. Among DNA or histone modifications, the repressive mark H3K27me3 is best characterized by its key roles in the control of cellular differentiation and reprogramming (Ikeuchi *et al.*, 2015b). POLYCOMB REPRESSIVE COMPLEX 2 (PRC2) is responsible for the deposition of H3K27me3 and maintains the repressed status of target loci. Genetic evidence showed that PRC2 is required for the maintenance of differentiated status in mature cells by preventing the ectopic onset of cellular de‐differentiation (Ikeuchi *et al.*, 2015a; Mozgová *et al.*, 2017) and may prevent hazardous cellular reprogramming within the context of tissue development. A longstanding question is how H3K27me3‐targeted genes become reactivated during cellular reprogramming. As mentioned above, the transcription factor STEMIN1 controls removal of H3K27me3 and concomitant induction of *CYCD;1* expression in *P. patens* (Ishikawa *et al.*, 2019) (Figure 2a). Detailed mechanisms of how STEMIN1 modifies histone marks within the target loci still remain unclear; therefore, we are examining various possibilities such as histone demethylase‐mediated regulation. Yan *et al*. (2020) proposed another mechanism for depleting repressive histone marks: H3K27me3‐marked histones are replaced by the histone variant H3.15, which is not targeted by PRC2, thereby evading polycomb group (PcG)‐mediated repression and enabling cellular reprogramming. Our project aims to evaluate the potential contribution of these mechanisms to cellular reprogramming.

To fully understand the epigenetic regulation of plant stem cells, it is imperative to unravel their behavior at high spatiotemporal resolution. Our project is developing single‐cell technologies, including single‐cell ATAC‐seq combined with fate tracking, which will bring new insights into epigenetic regulation underpinning the heterogeneity, robustness, and stochasticity in stem cell behavior.

## HORMONAL REGULATION OF STEM CELLS

In the shoot apex, stem cells are maintained in the indeterminate meristem, which continuously produces aboveground organs. Stem cells reside in the uppermost region of the dome‐shaped SAM. Research using Arabidopsis revealed that the formation and maintenance of the stem cell niche are supported by a robust system of mutual regulation of the homeodomain transcription factor WUSCHEL (WUS), the transmembrane receptor kinase CLAVATA1 (CLV1), and its ligand CLV3 (Fuchs and Lohmann, 2020; Han *et al.*, 2020). Cytokinin plays a pivotal role in the maintenance of the WUS/CLV circuit: for instance, it upregulates WUS function in multiple ways at the transcriptional and post‐translational levels (Fuchs and Lohmann, 2020; Lee *et al.*, 2019a; Snipes *et al.*, 2018). Previous studies suggest that little *de novo* cytokinin synthesis occurs in the SAM because biosynthetic gene expression is barely detectable (Kiba *et al.*, 2013; Yadav *et al.*, 2014) and that the majority of cytokinin acting in the SAM is supplied from other parts as a precursor (Osugi *et al.*, 2017). However, the dynamics and molecular mechanisms of cytokinin migration within the SAM, which should affect stem cell behavior, have not yet been well clarified. A key finding was that genes encoding LONELY GUY (LOG), which catalyzes the final step of cytokinin synthesis, are expressed in limited cell layers in the uppermost part of the SAM; specifically, *LOG4* is expressed in the L1 layer and *LOG7* in the central zone (Chickarmane *et al.*, 2012; Yadav *et al.*, 2009, 2014). Loss of their gene function affects traits associated with SAM activity, leading to a shortening of the plastochron (Tokunaga *et al.*, 2012). Grafting experiments using cytokinin‐related mutants showed that, although both the active form and the precursor (riboside form) are translocated to the shoot apex via the vascular bundles, cytokinin activity in the SAM is LOG‐dependent and requires precursor‐derived cytokinin (Osugi *et al.*, 2017). Therefore, a critical outstanding question is how precursors migrate to the top of the SAM where LOG is expressed.

The expression patterns of *CYTOKININ OXIDASE* (*CKX*), encoding the cytokinin‐degrading enzyme (Bartrina *et al.*, 2011; Yadav *et al.*, 2009), and the transporter gene *PURINE PERMEASE14* (*PUP14*), which decreases cytokinin in the apoplast (Zürcher *et al.*, 2016), suggest that the cytokinin concentration is tightly regulated in the stem cell niche. As described above, LOG functions in the uppermost layer of the SAM, while cytokinin receptor genes *ARABIDOPSIS HISTIDINE KINASE2* (*AHK2*), *AHK3*, and *AHK4*/*CRE1* are expressed in the organizing center, indicating that tissues for cytokinin production and perception are spatially separated, as the CLV3–CLV1 ligand–receptor system is. Therefore, another key question is whether cytokinin migration from the outer to the inner layers of the SAM occurs via passive diffusion or by means of an uncharacterized transport system. Our plant stem cell project aims to elucidate the machineries controlling cytokinin flow in the SAM, the apical transport of cytokinin precursors, and the flow down to the receptor after activation by LOG.

Auxin is also involved in the maintenance of the WUS/CLV circuit. Auxin affects cytokinin signaling by repressing the expression of *ARABIDOPSIS RESPONSE REGULATOR*s (*ARR7* and *ARR15*) and by inducing the *ARABIDOPSIS HISTIDINE PHOSPHOTRANSFER PROTEIN* (*AHP6*) gene via MONOPTEROS (MP)/ARF5 (Besnard *et al.*, 2014; Zhao *et al.*, 2010). MP/ARF5 also represses the expression of *DORNRÖSCHEN* (*DRN*)/*ENHANCER OF SHOOT REGENERATION 1* (*ESR1*) to promote *CLV3* expression (Luo *et al.*, 2018). Additionally, several genes involved in cytokinin biosynthesis and transport are known to be regulated by auxin, although how such crosstalk is associated with SAM activity remains elusive. A complete picture of the inter‐regulation of the two major hormones in the SAM will shed light on the molecular basis for the maintenance of the stem cell niche.

## TERMINATION OF STEM CELLS

Once the SAM is specified as a floral meristem, its activity stops after a defined number of floral organs are produced. The elaborate mechanisms to terminate stem cell activity in the floral meristem have been elucidated in recent studies. For floral meristem determinacy, the C‐class MADS‐domain transcription factors play a major role (Bowman *et al.*, 1989; Ito *et al.*, 2004; Yanofsky *et al.*, 1990). AGAMOUS (AG) directly and indirectly represses the key stem cell determinant gene *WUS* through multi‐step processes in Arabidopsis (Laux *et al.*, 1996; Liu *et al.*, 2011; Sun *et al.*, 2009). AG protein directly binds to the *WUS* promoter and gradually represses *WUS* expression by changing the epigenetic status through recruitment of PcG (Guo *et al.*, 2018; Liu *et al.*, 2011). In addition, AG directly induces *KNUCKLES (KNU)*, which encodes a C2H2 zinc finger protein, to fully shut down *WUS* when the appropriate number of cells have been produced (Payne *et al.*, 2004; Sun *et al.*, 2014; Sun *et al.*, 2009). We found that the binding of KNU to the *WUS* locus causes the eviction of a SWI/SNF chromatin remodeling factor and the recruitment of PcG onto the *WUS* promoter for stable silencing of *WUS* (Sun *et al.*, 2019). Furthermore, when *WUS* expression is terminated, AG induces *CRABS CLAW (CRC)*, a YABBY‐type transcription factor, at the abaxial side of the carpel primordia (Alvarez and Smyth, 1999; Bowman and Smyth, 1999; Yamaguchi *et al.*, 2017). CRC regulates auxin biosynthesis and transport as a failsafe mechanism that prevents overproliferation of stem cells when *KNU* is mutated (Yamaguchi *et al.*, 2018; Yamaguchi *et al.*, 2017).

In parallel to the AG pathway, the zinc finger transcription factor SUPERMAN (SUP) is induced around the same developmental stage as the increase of *AG* transcripts. The induction is observed in cells surrounding the stem cell population (Bowman *et al.*, 1992; Sakai *et al.*, 2000). We revealed that SUP negatively regulates auxin biosynthesis genes to prevent overgrowth of the stem cell population through PcG recruitment (Xu *et al.*, 2018). Moreover, the cytokinin signaling inhibitor *AHP6* is upregulated in the *sup crc* double mutant (Lee *et al.*, 2019b). These observations demonstrate that multiple transcription factors cooperatively act on hormone synthesis and signaling genes as well as *WUS* to shut off stem cell activities, thereby controlling floral meristem determinacy. As with the maintenance of the SAM, how auxin and cytokinin spatially and temporally regulate floral stem cell activities remains to be solved. Our project aims to describe hormone synthesis and signaling at a high spatiotemporal resolution and their effects on physiological and mechanical properties of floral stem cells.

Temporal regulation of the transition from the indeterminate to the determinate stage influences the inflorescence architecture, which is one of the major determinants of yield in seed harvesting crops. Therefore, genes involved in the phase change have been extensively studied by mutant screening, quantitative trait locus analysis, and genome‐wide association studies in crop species such as rice, maize (*Zea mays*), and tomato (*Solanum lycopersicum*) (Bommert and Whipple, 2018). *ABERRANT PANICLE ORGANIZATION1/2*, *TAWAWA1*, and *FRIZZY PANICLE* genes, encoding transcription factors, were isolated as regulators of the inflorescence form in rice (Ikeda‐Kawakatsu *et al.*, 2009; Komatsu *et al.*, 2003; Yoshida *et al.*, 2013). We discovered that these genes function through the control of stem cell activities in a partially overlapping manner. Our project will contribute not only to deepening our understanding of plant stem cells but also to providing basic knowledge to meet global food demand under the pressure of climate change and population growth.

## ASYMMETRIC DIVISION OF STEM CELLS

Stem cells undergo asymmetric division, after which one daughter cell remains a stem cell while the other begins to differentiate. Asymmetric division is therefore required for stem cell maintenance, but also for *de novo* stem cell generation: for example, in the stomatal lineage, asymmetric division from a protodermal cell named the meristemoid mother cell produces a meristemoid with a stem cell character and the other daughter cell that repeats asymmetric division to generate further meristemoids, which finally differentiate into guard cells and differentiated epidermal cells (Han and Torii, 2019).

Asymmetric division often accompanies daughter cell size asymmetry and/or asymmetric distribution of cytoplasmic/cortical fate determinants. In animal cells, this is largely controlled by proper localization and orientation of the mitotic spindle, which dictates the division site and orientation (Knoblich, 2010). The organizing force is generated by microtubules emanating outward from the centrosome (called astral microtubules), which are pulled by cortically attached dynein motors (Kiyomitsu, 2015). Asymmetry of cortical dynein ensures unidirectional motility of the spindle. In contrast, plants lack dynein motors as well as centrosomes, and so also lack prominent astral microtubules. Thus, a different mechanism must exist to perform asymmetric division. Until recently, the prevailing model for controlling spindle location and orientation involved microtubule arrays called the preprophase band (PPB), which appears prior to mitosis beneath the cell cortex and around the nucleus. The PPB ensures the bipolarity of prophase spindles and forecasts future division sites, and therefore had been proposed as a centrosome analog (Rasmussen *et al.*, 2013). However, Schaefer et al. (2017) challenged this view: an Arabidopsis mutant that specifically abolishes PPB formation grows normally overall, with only minor loss of growth capacity and developmental robustness.

Our project has been tackling the mechanism of asymmetric stem cell division mainly in the moss *P. patens*, which naturally lacks PPBs in most tissues. Thus far, we have discovered three important elements that dictate spatial control of the division plane. One is nuclear positioning (Figure 2b). In protonemal stem cells, endoplasmic microtubules and microtubule‐based kinesin motors are required for nuclear positioning in the interphase cytoplasm. In the absence of the retrograde transporter KCH or the anterograde motor kinesin‐ARK, the spindle position is skewed, resulting in an abnormal daughter cell size ratio (Miki *et al.*, 2015; Yamada and Goshima, 2018). Similarly, in Arabidopsis zygotes, nuclear positioning plays a key role in division site determination (Kimata *et al.*, 2019). In both systems, cell polarization is a cue to trigger a change in microtubule polarity, which leads to directional nuclear motility in the cytoplasm (Kimata *et al.*, 2016; Yi and Goshima, 2020). A similar scheme, involving polarity establishment and nuclear positioning, has been identified in asymmetric cell division during lateral root initiation and in the stomatal lineage in Arabidopsis (Muroyama *et al.*, 2020; Vilches Barro *et al.*, 2019). Interestingly, in Arabidopsis zygotes, misplacement of the vacuole affects nuclear position and division site, suggesting a link between vacuolar dynamics and asymmetric cell division (Kimata *et al.*, 2019).

The second element is microtubule structures that are functionally analogous to the centrosome. We found that in the moss gametophore initial cell, a cloud of microtubules, termed the gametosome, appears transiently in the prophase cytoplasm and works as the dominant microtubule organizing center required for spindle orientation (Figure 2b) (Kosetsu *et al.*, 2017). A similar structure, called the polar organizer (PO), is observed during mitosis in liverwort (*Marchantia polymorpha*) cells (Buschmann *et al.*, 2016). In seed plants, polar caps (also called prospindles) that surround the prophase nucleus have a similar function (Figure 2b); pharmacological inhibition of polar cap formation did not prevent spindle formation but skewed its orientation (Kosetsu *et al.*, 2017). Currently, the mechanisms of gametosome/PO/prospindle formation are largely unknown, other than the essential contribution of γ‐tubulin‐dependent microtubule nucleation (Yi and Goshima, 2018).

Finally, recent data indicate that metaphase spindles can be mobile, like animal spindles. In a mutant of the microtubule‐associated protein TPX2 in *P. patens*, the bipolar spindle in the gametophore stem cells moved basally, such that the division plane was dramatically skewed (Kozgunova *et al.*, 2020). Interestingly, this motility was completely suppressed by actin inhibition. Critical involvement of actin in spindle positioning is characteristic of animal oocytes, which lack asters (Uraji *et al.*, 2018). Therefore, our observation in moss raises the possibility that plants have developed a similar mechanism to animal oocytes. In coming years, we aim to uncover the key processes of asymmetric division in plants and compare them with those of animals, and thereby to understand the mechanisms underlying stem cell maintenance and generation in a developmental context.

## GENOME STABILITY OF STEM CELLS

Plant lifespan is characterized by a rudimentary body plan, modular growth, and disparity between cell death and death of the organism (Watson and Riha, 2011). Plants exhibit a wide range of lifespans from a few weeks in monocarpic annuals to as long as millennia in long‐lived perennials, which harbor meristematic cells that undergo thousands of divisions. In addition, plants being sessile organisms, environmental stresses increase DNA damage in stem cells; therefore, how efficient the DNA repair mechanisms are in long‐lived plant species and what the difference is between repair mechanisms in plants and animals are interesting questions to be answered.

Previous work focusing on animal aging highlighted the positive correlation between increased copy number of DNA repair genes and longevity in mammals (Tian *et al.*, 2017). The naked mole‐rat, the longest‐lived rodent with a maximum lifespan of 32 years, has a higher copy number of genes for CCAAT/enhancer binding protein‐γ (CEBPG), a regulator of DNA repair, and TERF1‐interacting nuclear factor 2 (TINF2), a protector of telomere integrity, than short‐lived rodent species (MacRae *et al.*, 2015). Another long‐lived mammal, the African elephant, encodes 20 copies of the tumor suppressor gene *TP53*, which induces apoptosis or senescence programs in response to DNA damage (Sulak *et al.*, 2016). Analyses of genomes of two other long‐lived species, the bowhead whale and bat, showed the signature of positive selection of multiple DNA repair and DNA damage‐signaling genes (Keane *et al.*, 2015; Zhang *et al.*, 2013). These reports suggest the importance of genome maintenance mechanisms for longevity. However, in plants, no studies have yet employed comparative genome analyses to identify DNA repair genes associated with the evolution of longevity. Thanks to substantial progress in the elucidation of DNA damage signaling and repair mechanisms in Arabidopsis (Manova and Gruszka, 2015), it has become evident that most of the major DNA repair pathways are conserved in plants. Our plant stem cell project aims to systematically compare the DNA repair systems of diverse plant species and uncover their effects on organismal phenotypes such as mutation rates, lifespan, and adaptation to extreme environments, thereby identifying the role of DNA repair mechanisms in stem cell maintenance.

In Arabidopsis, stem cells highly express DNA repair genes, such as *RADIATION SENSITIVE 51* (*RAD51*) and *BREAST CANCER SUSCEPTIBILITY 1* (*BRCA1*), which maintain genome integrity (Yadav *et al.*, 2009). However, severe DNA damage induces selective death of stem cells, but not of other somatic cells, in a programmed manner, and stem cells are replenished by activation of cell division in the adjacent organizing center (Fulcher and Sablowski, 2009; Furukawa *et al.*, 2010). In mammals, cell death induction is a common strategy to cope with DNA damage, suggesting that plants trigger cell death in a stem cell‐specific manner to prioritize the avoidance of unexpected destruction of developing tissues caused by disordered cell death. In spite of such a unique feature, information about stem cell death in plants is fragmentary: DNA damage‐induced cell death is suppressed in Arabidopsis mutants of the brassinosteroid receptor BRI1 and the transcription factors ANAC044 and ANAC085, which are involved in cell cycle arrest (Chen *et al.*, 2017; Lozano‐Elena *et al.*, 2018; Takahashi *et al.*, 2019), although the link between brassinosteroid signaling and the cell cycle remains elusive. By contrast, the mechanism of stem cell replenishment has been uncovered in a recent study of the root stem cell niche; the transcription factor ERF115, which is induced by brassinosteroid, promotes quiescent center cell division, thereby providing new stem cells after DNA damage (Heyman *et al.*, 2013). Interestingly, ERF115 also triggers cell division adjacent to collapsed differentiated cells in roots (Canher *et al.*, 2020; Heyman *et al.*, 2016), suggesting that an ERF115‐mediated pathway is a common system promoting cell division next to dead cells and regenerating tissues. Our focus is on how stem cell replenishment is fine‐tuned to properly reconstitute the stem cell niche and how genome stability is preserved in stem cells. By answering these questions, we will better understand how plant longevity is guaranteed under fluctuating environmental conditions and what its essential difference is from animals.

## CONCLUDING THOUGHTS

Outstanding questions in our research field include how plant stem cells maintain pluripotency throughout life and what determines the initial step of reprogramming and stem cell formation. Accumulating evidence in our group project highlights the importance of cell‐to‐cell communication in both stem cell initiation and maintenance. Phytohormones seem to play a major role, and their crosstalk is absolutely crucial for defining stem cells, while how their signaling controls chromatin status remains largely unknown. Recent advances in single‐cell analysis and hormone detection will open the way to a full understanding of stem cells’ behavior and their response to environmental inputs. Eventually, uncovering the secrets of plant stem cells will pave the way for developing new technologies that increase plant productivity and preserve plant species diversity and will provide clues to overcome food supply and environmental problems.

## CONFLICT OF INTEREST

The authors have no conflict of interest to declare.

## References

[tpj15184-bib-0001] Alvarez, J. & Smyth, D.R. (1999) CRABS CLAW and SPATULA, two *Arabidopsis* genes that control carpel development in parallel with AGAMOUS. Development, 126, 2377–2386. 10225997 10.1242/dev.126.11.2377

[tpj15184-bib-0002] Bartrina, I. , Otto, E. , Strnad, M. , Werner, T. & Schmülling, T. (2011) Cytokinin regulates the activity of reproductive meristems, flower organ size, ovule formation, and thus seed yield in *Arabidopsis thaliana* . The Plant Cell, 23, 69–80. 21224426 10.1105/tpc.110.079079 PMC3051259

[tpj15184-bib-0003] Besnard, F. , Refahi, Y. , Morin, V. , Marteaus, B. , Brunoud, G. ***et al***. (2014) Cytokinin signalling inhibitory fields provide robustness to phyllotaxis. Nature, 505, 417–421. 24336201 10.1038/nature12791

[tpj15184-bib-0004] Birnbaum, K.D. (2016) How many ways are there to make a root? Current Opinion in Plant Biology, 34, 61–67. 27780106 10.1016/j.pbi.2016.10.001

[tpj15184-bib-0005] Birnbaum, K.D. & Sanchez Alvarado, A. (2008) Slicing across kingdoms: regeneration in plants and animals. Cell, 132, 697–710. 18295584 10.1016/j.cell.2008.01.040 PMC2692308

[tpj15184-bib-0006] Bommert, P. & Whipple, C. (2018) Grass inflorescence architecture and meristem determinacy. Seminars in Cell & Developmental Biology, 79, 37–47. 29020602 10.1016/j.semcdb.2017.10.004

[tpj15184-bib-0007] Bowman, J.L. , Sakai, H. , Jack, T. , Weigel, D. , Mayer, U. & Meyerowitz, E.M. (1992) SUPERMAN, a regulator of floral homeotic genes in *Arabidopsis* . Development, 114, 599–615. 1352237 10.1242/dev.114.3.599

[tpj15184-bib-0008] Bowman, J.L. & Smyth, D.R. (1999) CRABS CLAW, a gene that regulates carpel and nectary development in *Arabidopsis*, encodes a novel protein with zinc finger and helix‐loop‐helix domains. Development, 126, 2387–2396. 10225998 10.1242/dev.126.11.2387

[tpj15184-bib-0009] Bowman, J.L. , Smyth, D.R. & Meyerowitz, E.M. (1989) Genes directing flower development in *Arabidopsis* . The Plant Cell, 1, 37–52. 2535466 10.1105/tpc.1.1.37 PMC159735

[tpj15184-bib-0010] Bridgen, M.P. , Hadi, M.Z. & Spencer‐Barreto, M. (1994) A laboratory exercise to demonstrate direct and indirect shoot organogenesis from leaves of *Torenia fournieri* . Horttechnology, 4, 320–322.

[tpj15184-bib-0011] Buschmann, H. , Holtmannspotter, M. , Borchers, A. , O'Donoghue, M.T. & Zachgo, S. (2016) Microtubule dynamics of the centrosome‐like polar organizers from the basal land plant *Marchantia polymorpha* . New Phytologist, 209, 999–1013. 10.1111/nph.13691 26467050

[tpj15184-bib-0012] Canher, B. , Heyman, J. , Savina, M. , Devendran, A. , Eekhout, T. , Vercauteren, I. ***et al***. (2020) Rocks in the auxin stream: Wound‐induced auxin accumulation and *ERF115* expression synergistically drive stem cell regeneration. Proceedings of the National Academy of Sciences of the United States of America, 117, 16667–16677. 32601177 10.1073/pnas.2006620117 PMC7368246

[tpj15184-bib-0013] Che, P. , Lall, S. , Nettleton, D. & Howell, S.H. (2006) Gene expression programs during shoot, root, and callus development in *Arabidopsis* tissue culture. Plant Physiology, 141, 620–637. 16648215 10.1104/pp.106.081240 PMC1475446

[tpj15184-bib-0014] Chen, P. , Takatsuka, H. , Takahashi, N. , Kurata, R. , Fukao, Y. , Kobayashi, K. ***et al***. (2017) *Arabidopsis* R1R2R3‐Myb proteins are essential for inhibiting cell division in response to DNA damage. Nature Communications, 8, 635. 10.1038/s41467-017-00676-4 PMC5608833 28935922

[tpj15184-bib-0015] Chickarmane, V.S. , Gordon, S.P. , Tarr, P.T. , Heisler, M.G. & Meyerowitz, E.M. (2012) Cytokinin signaling as a positional cue for patterning the apical‐basal axis of the growing *Arabidopsis* shoot meristem. Proceedings of the National Academy of Sciences of the United States of America, 109, 4002–4007. 22345559 10.1073/pnas.1200636109 PMC3309735

[tpj15184-bib-0016] Deal, R.B. & Henikoff, S. (2010) A simple method for gene expression and chromatin profiling of individual cell types within a tissue. Developmental Cell, 18, 1030–1040. 20627084 10.1016/j.devcel.2010.05.013 PMC2905389

[tpj15184-bib-0017] Ferguson, B.J. , Mens, C. , Hastwell, A.H. , Zhang, M. , Su, H. , Jones, C.H. ***et al***. (2019) Legume nodulation: the host controls the party. Plant, Cell and Environment, 42, 41–51. 10.1111/pce.13348 29808564

[tpj15184-bib-0018] Fuchs, M. & Lohmann, J.U. (2020) Aiming for the top: non‐cell autonomous control of shoot stem cells in *Arabidopsis* . Journal of Plant Research, 133, 297–309. 32146616 10.1007/s10265-020-01174-3 PMC7214502

[tpj15184-bib-0019] Fulcher, N. & Sablowski, R. (2009) Hypersensitivity to DNA damage in plant stem cell niches. Proceedings of the National Academy of Sciences of the United States of America, 106, 20984–20988. 19933334 10.1073/pnas.0909218106 PMC2791609

[tpj15184-bib-0020] Furukawa, T. , Curtis, M.J. , Tominey, C.M. , Duong, Y.H. , Wilcox, B.W.L. , Aggoune, D. ***et al***. (2010) A shared DNA‐damage‐response pathway for induction of stem‐cell death by UVB and by gamma irradiation. DNA Repair, 9, 940–948. 20634150 10.1016/j.dnarep.2010.06.006

[tpj15184-bib-0021] Gu, N. , Tamada, Y. , Imai, A. , Palfalvi, G. , Kabeya, Y. , Shigenobu, S. ***et al***. (2020) DNA damage triggers reprogramming of differentiated cells into stem cells in *Physcomitrella* . Nature Plant, 6, 1098–1105. 10.1038/s41477-020-0745-9 32807952

[tpj15184-bib-0022] Guo, L. , Cao, X. , Liu, Y. , Li, J. , Li, Y. , Li, D. ***et al***. (2018) A chromatin loop represses WUSCHEL expression in *Arabidopsis* . The Plant Journal, 94, 1083–1097. 29660180 10.1111/tpj.13921

[tpj15184-bib-0023] Gutzat, R. , Rembart, K. , Nussbaumer, T. , Hofmann, F. , Pisupati, R. ***et al***. (2020) *Arabidopsis* shoot stem cells display dynamic transcription and DNA methylation patterns. EMBO Journal, 39, e103667. 10.15252/embj.2019103667 PMC7560203 32815560

[tpj15184-bib-0024] Han, H. , Liu, X. & Zhou, Y. (2020) Transcriptional circuits in control of shoot stem cell homeostasis. Current Opinion in Plant Biology, 53, 50–56. 31766002 10.1016/j.pbi.2019.10.004

[tpj15184-bib-0025] Han, S.K. & Torii, K.U. (2019) Linking cell cycle to stomatal differentiation. Current Opinion in Plant Biology, 51, 66–73. 31075538 10.1016/j.pbi.2019.03.010

[tpj15184-bib-0026] Heyman, J. , Cools, T. , Canher, B. , Shavialenka, S. , Traas, J. , Vercauteren, I. ***et al***. (2016) The heterodimeric transcription factor complex ERF115‐PAT1 grants regeneration competence. Nature Plant, 2, 16165. 10.1038/nplants.2016.165 27797356

[tpj15184-bib-0027] Heyman, J. , Cools, T. , Vandenbussche, F. , Heyndrickx, K.S. , Van Leene, J. , Vercauteren, I. ***et al***. (2013) ERF115 controls root quiescent center cell division and stem cell replenishment. Science, 342, 860–863. 24158907 10.1126/science.1240667

[tpj15184-bib-0028] Higo, A. , Saihara, N. , Miura, F. , Higashi, Y. , Yamada, M. ***et al***. (2020) DNA methylation is reconfigured at the onset of reproduction in rice shoot apical meristem. Nature Communications, 11, 4079. 10.1038/s41467-020-17963-2 PMC7429860 32796936

[tpj15184-bib-0029] Ichihashi, Y. , Hakoyama, T. , Iwase, A. , Shirasu, K. , Sugimoto, K. & Hayashi, M. (2020) Common mechanisms of developmental reprogramming in plants‐lessons from regeneration, symbiosis, and parasitism. Frontiers in Plant Science, 11, 1084. 32765565 10.3389/fpls.2020.01084 PMC7378864

[tpj15184-bib-0030] Ikeda‐Kawakatsu, K. , Yasuno, N. , Oikawa, T. , Iida, S. , Nagato, Y. , Maekawa, M. ***et al***. (2009) Expression level of ABERRANT PANICLE ORGANIZATION1 determines rice inflorescence form through control of cell proliferation in the meristem. Plant Physiology, 150, 736–747. 19386809 10.1104/pp.109.136739 PMC2689948

[tpj15184-bib-0031] Ikeuchi, M. , Favero, D.S. , Sakamoto, Y. , Iwase, A. , Coleman, D. , Rymen, B. ***et al***. (2019) Molecular mechanisms of plant regeneration. Annual Review of Plant Biology, 70, 377–406. 10.1146/annurev-arplant-050718-100434 30786238

[tpj15184-bib-0032] Ikeuchi, M. , Iwase, A. , Rymen, B. , Harashima, H. , Shibata, M. ***et al***. (2015a) PRC2 represses dedifferentiation of mature somatic cells in *Arabidopsis* . Nature Plant, 1, 15089. 10.1038/nplants.2015.89 27250255

[tpj15184-bib-0033] Ikeuchi, M. , Iwase, A. , Rymen, B. , Lambolez, A. , Kojima, M. , Takebayashi, Y. ***et al***. (2017) Wounding triggers callus formation via dynamic hormonal and transcriptional changes. Plant Physiology, 175, 1158–1174. 28904073 10.1104/pp.17.01035 PMC5664475

[tpj15184-bib-0034] Ikeuchi, M. , Iwase, A. & Sugimoto, K. (2015b) Control of plant cell differentiation by histone modification and DNA methylation. Current Opinion in Plant Biology, 28, 60–67. 26454697 10.1016/j.pbi.2015.09.004

[tpj15184-bib-0035] Ishikawa, M. , Morishita, M. , Higuchi, Y. , Ichikawa, S. , Ishikawa, T. , Nishiyama, T. ***et al***. (2019) Physcomitrella STEMIN transcription factor induces stem cell formation with epigenetic reprogramming. Nature Plant, 5, 681–690. 10.1038/s41477-019-0464-2 31285563

[tpj15184-bib-0036] Ishikawa, M. , Murata, T. , Sato, Y. , Nishiyama, T. , Hiwatashi, Y. , Imai, A. ***et al***. (2011) *Physcomitrella* cyclin‐dependent kinase A links cell cycle reactivation to other cellular changes during reprogramming of leaf cells. The Plant Cell, 23, 2924–2938. 21862705 10.1105/tpc.111.088005 PMC3180801

[tpj15184-bib-0037] Ito, T. , Wellmer, F. , Yu, H. , Das, P. , Ito, N. , Alves‐Ferreira, M. ***et al***. (2004) The homeotic protein AGAMOUS controls microsporogenesis by regulation of SPOROCYTELESS. Nature, 430, 356–360. 15254538 10.1038/nature02733

[tpj15184-bib-0038] Iwafuchi‐Doi, M. & Zaret, K.S. (2014) Pioneer transcription factors in cell reprogramming. Genes & Development, 28, 2679–2692. 25512556 10.1101/gad.253443.114 PMC4265672

[tpj15184-bib-0039] Iwase, A. , Mitsuda, N. , Koyama, T. , Hiratsu, K. , Kojima, M. , Arai, T. ***et al***. (2011) The AP2/ERF transcription factor WIND1 controls cell dedifferentiation in *Arabidopsis* . Current Biology, 21, 508–514. 21396822 10.1016/j.cub.2011.02.020

[tpj15184-bib-0040] Keane, M. , Semeiks, J. , Webb, A.E. , Li, Y.I. , Quesada, V. ***et al***. (2015) Insights into the evolution of longevity from the bowhead whale genome. Cell Reports, 10, 112–122. 25565328 10.1016/j.celrep.2014.12.008 PMC4536333

[tpj15184-bib-0041] Kiba, T. , Takei, K. , Kojima, M. & Sakakibara, H. (2013) Side‐chain modification of cytokinins controls shoot growth in *Arabidopsis* . Developmental Cell, 27, 452–461. 24286826 10.1016/j.devcel.2013.10.004

[tpj15184-bib-0042] Kimata, Y. , Higaki, T. , Kawashima, T. , Kurihara, D. , Sato, Y. , Yamada, T. ***et al***. (2016) Cytoskeleton dynamics control the first asymmetric cell division in *Arabidopsis* zygote. Proceedings of the National Academy of Sciences of the United States of America, 113, 14157–14162. 27911812 10.1073/pnas.1613979113 PMC5150365

[tpj15184-bib-0043] Kimata, Y. , Kato, T. , Higaki, T. , Kurihara, D. , Yamada, T. , Segami, S. ***et al***. (2019) Polar vacuolar distribution is essential for accurate asymmetric division of *Arabidopsis* zygotes. Proceedings of the National Academy of Sciences of the United States of America, 116, 2338–2343. 30651313 10.1073/pnas.1814160116 PMC6369786

[tpj15184-bib-0044] Kiyomitsu, T. (2015) Mechanisms of daughter cell‐size control during cell division. Trends in Cell Biology, 25, 286–295. 25548067 10.1016/j.tcb.2014.12.003

[tpj15184-bib-0045] Knoblich, J.A. (2010) Asymmetric cell division: recent developments and their implications for tumour biology. Nature Reviews Molecular Cell Biology, 11, 849–860. 21102610 10.1038/nrm3010 PMC3941022

[tpj15184-bib-0046] Komatsu, M. , Chujo, A. , Nagato, Y. , Shimamoto, K. & Kyozuka, J. (2003) FRIZZY PANICLE is required to prevent the formation of axillary meristems and to establish floral meristem identity in rice spikelets. Development, 130, 3841–3850. 12835399 10.1242/dev.00564

[tpj15184-bib-0047] Komiya, R. , Ikegami, A. , Tamaki, S. , Yokoi, S. & Shimamoto, K. (2008) *Hd3a* and *RFT1* are essential for flowering in rice. Development, 135, 767–774. 18223202 10.1242/dev.008631

[tpj15184-bib-0048] Kosetsu, K. , Murata, T. , Yamada, M. , Nishina, M. , Boruc, J. , Hasebe, M. ***et al***. (2017) Cytoplasmic MTOCs control spindle orientation for asymmetric cell division in plants. Proceedings of the National Academy of Sciences of the United States of America, 114, E8847–E8854. 28973935 10.1073/pnas.1713925114 PMC5651782

[tpj15184-bib-0049] Kozgunova, E. , Yoshida, M.W. & Goshima, G. (2020) Spindle position dictates division site during asymmetric cell division in moss. bioRxiv. 10.1101/2020.03.03.975557 PMC9072379 35513464

[tpj15184-bib-0050] Laux, T. , Mayer, K.F. , Berger, J. & Jurgens, G. (1996) The WUSCHEL gene is required for shoot and floral meristem integrity in *Arabidopsis* . Development, 122, 87–96. 8565856 10.1242/dev.122.1.87

[tpj15184-bib-0051] Lee, Z.H. , Hirakawa, T. , Yamaguchi, N. & Ito, T. (2019a) The roles of plant hormones and their interactions with regulatory genes in determining meristem activity. International Journal of Molecular Sciences, 20, 4065. 10.3390/ijms20164065 PMC6720427 31434317

[tpj15184-bib-0052] Lee, Z.H. , Tatsumi, Y. , Ichihashi, Y. , Suzuki, T. , Shibata, A. , Shirasu, K. ***et al***. (2019b) CRABS CLAW and SUPERMAN coordinate hormone‐, stress‐, and metabolic‐related gene expression during *Arabidopsis* stamen development. Frontiers in Ecology and Evolution, 7, 437.

[tpj15184-bib-0053] Liu, X. , Kim, Y.J. , Muller, R. , Yumul, R.E. , Liu, C. , Pan, Y. ***et al***. (2011) AGAMOUS terminates floral stem cell maintenance in Arabidopsis by directly repressing WUSCHEL through recruitment of Polycomb Group proteins. The Plant Cell, 23, 3654–3670. 22028461 10.1105/tpc.111.091538 PMC3229141

[tpj15184-bib-0054] Lozano‐Elena, F. , Planas‐Riverola, A. , Vilarrasa‐Blasi, J. , Schwab, R. & Caño‐Delgado, A.I. (2018) Paracrine brassinosteroid signaling at the stem cell niche controls cellular regeneration. Journal of Cell Science, 131, jcs204065. 29242230 10.1242/jcs.204065 PMC5818034

[tpj15184-bib-0055] Luo, L. , Zeng, J. , Wu, H. , Tian, Z. & Zhao, Z. (2018) A molecular framework for auxin‐controlled homeostasis of shoot stem cells in *Arabidopsis* . Molecular Plant, 11, 899–913. 29730265 10.1016/j.molp.2018.04.006

[tpj15184-bib-0056] MacRae, S.L. , Croken, M.M. , Calder, R.B. , Aliper, A. , Milholland, B. ***et al***. (2015) DNA repair in species with extreme lifespan differences. Aging, 7, 1171. 26729707 10.18632/aging.100866 PMC4712340

[tpj15184-bib-0057] Manova, V. & Gruszka, D. (2015) DNA damage and repair in plants–from models to crops. Frontiers in Plant Science, 6, 885. 26557130 10.3389/fpls.2015.00885 PMC4617055

[tpj15184-bib-0058] Miki, T. , Nishina, M. & Goshima, G. (2015) RNAi screening identifies the armadillo repeat‐containing kinesins responsible for microtubule‐dependent nuclear positioning in *Physcomitrella patens* . Plant and Cell Physiology, 56, 737–749. 25588389 10.1093/pcp/pcv002

[tpj15184-bib-0059] Mozgová, I. , Muñoz‐Viana, R. & Hennig, L. (2017) PRC2 represses hormone‐induced somatic embryogenesis in vegetative tissue of *Arabidopsis thaliana* . PLoS Genetics, 13, e1006562. 28095419 10.1371/journal.pgen.1006562 PMC5283764

[tpj15184-bib-0060] Muroyama, A. , Gong, Y. & Bergmann, D.C. (2020) Opposing, polarity‐driven nuclear migrations underpin asymmetric divisions to pattern *Arabidopsis* stomata. Current Biology, 30, 1–9. 33202223 10.1016/j.cub.2020.09.087 PMC7938707

[tpj15184-bib-0061] Osugi, A. , Kojima, M. , Takebayashi, Y. , Ueda, N. , Kiba, T. & Sakakibara, H. (2017) Systemic transport of trans‐zeatin and its precursor have differing roles in *Arabidopsis* shoots. Nature Plant, 3, 17112. 10.1038/nplants.2017.112 28737742

[tpj15184-bib-0062] Payne, T. , Johnson, S.D. & Koltunow, A.M. (2004) KNUCKLES (KNU) encodes a C2H2 zinc‐finger protein that regulates development of basal pattern elements of the *Arabidopsis* gynoecium. Development, 131, 3737–3749. 15240552 10.1242/dev.01216

[tpj15184-bib-0063] Rasmussen, C.G. , Wright, A.J. & Muller, S. (2013) The role of the cytoskeleton and associated proteins in determination of the plant cell division plane. The Plant Journal, 75, 258–269. 23496276 10.1111/tpj.12177

[tpj15184-bib-0064] Rymen, B. , Kawamura, A. , Lambolez, A. , Inagaki, S. , Takebayashi, A. , Iwase, A. ***et al***. (2019) Histone acetylation orchestrates wound‐induced transcriptional activation and cellular reprogramming in *Arabidopsis* . Communications Biology, 2, 404. 31701032 10.1038/s42003-019-0646-5 PMC6828771

[tpj15184-bib-0065] Sakai, H. , Krizek, B.A. , Jacobsen, S.E. & Meyerowitz, E.M. (2000) Regulation of SUP expression identifies multiple regulators involved in *Arabidopsis* floral meristem development. The Plant Cell, 12, 1607–1618. 11006335 10.1105/tpc.12.9.1607 PMC149073

[tpj15184-bib-0066] Schaefer, E. , Belcram, K. , Uyttewaal, M. , Duroc, Y. , Goussot, M. , Legland, D. ***et al***. (2017) The preprophase band of microtubules controls the robustness of division orientation in plants. Science, 356, 186–189. 28408602 10.1126/science.aal3016

[tpj15184-bib-0067] Sijacic, P. , Bajic, M. , McKinney, E.C. , Meagher, R.B. & Deal, R.B. (2018) Changes in chromatin accessibility between *Arabidopsis* stem cells and mesophyll cells illuminate cell type‐specific transcription factor networks. The Plant Journal, 94, 215–231. 29513366 10.1111/tpj.13882 PMC7219318

[tpj15184-bib-0068] Snipes, S.A. , Rodriguez, K. , DeVries, A.E. , Miyawaki, K.N. , Perales, M. , Xie, M. ***et al***. (2018) Cytokinin stabilizes WUSCHEL by acting on the protein domains required for nuclear enrichment and transcription. PLoS Genetics, 14, e1007351. 29659567 10.1371/journal.pgen.1007351 PMC5919686

[tpj15184-bib-0069] Sulak, M. , Fong, L. , Mika, K. , Chigurupati, S. , Yon, L. , Mongan, N.P. ***et al***. (2016) TP53 copy number expansion is associated with the evolution of increased body size and an enhanced DNA damage response in elephants. eLife, 5, e11994. 27642012 10.7554/eLife.11994 PMC5061548

[tpj15184-bib-0070] Sun, B. , Looi, L.S. , Guo, S. , He, Z. , Gan, E.S. , Huang, J. ***et al***. (2014) Timing mechanism dependent on cell division is invoked by Polycomb eviction in plant stem cells. Science, 343, 1248559. 24482483 10.1126/science.1248559

[tpj15184-bib-0071] Sun, B. , Xu, Y. , Ng, K.‐H. & Ito, T. (2009) A timing mechanism for stem cell maintenance and differentiation in the *Arabidopsis* floral meristem. Genes & Development, 23, 1791–1804. 19651987 10.1101/gad.1800409 PMC2720260

[tpj15184-bib-0072] Sun, B. , Zhou, Y. , Cai, J. , Shang, E. , Yamaguchi, N. , Xiao, J. ***et al***. (2019) Integration of transcriptional repression and Polycomb‐mediated silencing of *WUSCHEL* in floral meristems. The Plant Cell, 31, 1488–1505. 31068455 10.1105/tpc.18.00450 PMC6635863

[tpj15184-bib-0073] Takahashi, N. , Ogita, N. , Takahashi, T. , Taniguchi, S. , Tanaka, M. , Seki, M. ***et al***. (2019) A regulatory module controlling stress‐induced cell cycle arrest in Arabidopsis. eLife, 8, e43944. 30944065 10.7554/eLife.43944 PMC6449083

[tpj15184-bib-0074] Tian, X. , Seluanov, A. & Gorbunova, V. (2017) Molecular mechanisms determining lifespan in short‐and long‐lived species. Trends in Endocrinology and Metabolism, 28, 722–734. 28888702 10.1016/j.tem.2017.07.004 PMC5679293

[tpj15184-bib-0075] Tokunaga, H. , Kojima, M. , Kuroha, T. , Ishida, T. , Sugimoto, K. , Kiba, T. ***et al***. (2012) *Arabidopsis* lonely guy (LOG) multiple mutants reveal a central role of the LOG‐dependent pathway in cytokinin activation. The Plant Journal, 69, 355–365. 22059596 10.1111/j.1365-313X.2011.04795.x

[tpj15184-bib-0076] Uraji, J. , Scheffler, K. & Schuh, M. (2018) Functions of actin in mouse oocytes at a glance. Journal of Cell Science, 131, jcs218099. 30467138 10.1242/jcs.218099

[tpj15184-bib-0077] Vilches Barro, A. , Stockle, D. , Thellmann, M. , Ruiz‐Duarte, P. , Bald, L. , Louveaux, M. ***et al***. (2019) Cytoskeleton dynamics are necessary for early events of lateral root initiation in *Arabidopsis* . Current Biology, 29, 2443–2454. 31327713 10.1016/j.cub.2019.06.039

[tpj15184-bib-0078] Watson, J.M. & Riha, K. (2011) Telomeres, aging, and plants: from weeds to Methuselah – a mini‐review. Gerontology, 57, 129–136. 20375491 10.1159/000310174

[tpj15184-bib-0079] Xu, Y. , Prunet, N. , Gan, E.‐S. , Wang, Y. , Stewart, D. , Wellmer, F. ***et al***. (2018) SUPERMAN regulates floral whorl boundaries through control of auxin biosynthesis. EMBO Journal, 37, e97499. 10.15252/embj.201797499 PMC5983216 29764982

[tpj15184-bib-0080] Yadav, R.K. , Girke, T. , Pasala, S. , Xie, M. & Reddy, G.V. (2009) Gene expression map of the *Arabidopsis* shoot apical meristem stem cell niche. Proceedings of the National Academy of Sciences of the United States of America, 106, 4941–4946. 19258454 10.1073/pnas.0900843106 PMC2660727

[tpj15184-bib-0081] Yadav, R.K. , Tavakkoli, M. , Xie, M. , Girke, T. & Venugopala, R.G. (2014) A high‐resolution gene expression map of the *Arabidopsis* shoot meristem stem cell niche. Development, 141, 2735–2744. 24961803 10.1242/dev.106104

[tpj15184-bib-0082] Yamada, M. & Goshima, G. (2018) The KCH kinesin drives nuclear transport and cytoskeletal coalescence to promote tip cell growth in *Physcomitrella patens* . The Plant Cell, 30, 1496–1510. 29880712 10.1105/tpc.18.00038 PMC6096588

[tpj15184-bib-0083] Yamaguchi, N. , Huang, J. , Tatsumi, Y. , Abe, M. , Sugano, S.S. , Kojima, M. ***et al***. (2018) Chromatin‐mediated feed‐forward auxin biosynthesis in floral meristem determinacy. Nature Communications, 9, 5290. 10.1038/s41467-018-07763-0 PMC6289996 30538233

[tpj15184-bib-0084] Yamaguchi, N. , Huang, J. , Xu, Y. , Tanoi, K. & Ito, T. (2017) Fine‐tuning of auxin homeostasis governs the transition from floral stem cell maintenance to gynoecium formation. Nature Communications, 8, 1125. 10.1038/s41467-017-01252-6 PMC5654772 29066759

[tpj15184-bib-0085] Yan, A. , Borg, M. , Berger, F. & Chen, Z. (2020) The atypical histone variant H3.15 promotes callus formation in *Arabidopsis thaliana* . Development, 147, dev184895. 32439757 10.1242/dev.184895

[tpj15184-bib-0086] Yanofsky, M.F. , Ma, H. , Bowman, J.L. , Drews, G.N. , Feldmann, K.A. & Meyerowitz, E.M. (1990) The protein encoded by the *Arabidopsis* homeotic gene agamous resembles transcription factors. Nature, 346, 35–39. 1973265 10.1038/346035a0

[tpj15184-bib-0087] Yasui, Y. , Tsukamoto, S. , Sugaya, T. , Nishihama, R. , Wang, Q. , Kato, H. ***et al***. (2019) GEMMA CUP‐ASSOCIATED MYB1, an ortholog of axillary meristem regulators, is essential in vegetative reproduction in *Marchantia polymorpha* . Current Biology, 29, 3987–3995. 31708390 10.1016/j.cub.2019.10.004

[tpj15184-bib-0088] Yi, P. & Goshima, G. (2018) Microtubule nucleation and organization without centrosomes. Current Opinion in Plant Biology, 46, 1–7. 29981930 10.1016/j.pbi.2018.06.004

[tpj15184-bib-0089] Yi, P. & Goshima, G. (2020) Rho of plants GTPases and cytoskeletal elements control nuclear positioning and asymmetric cell division during *Physcomitrella patens* branching. Current Biology, 30, 2860–2868. 32470363 10.1016/j.cub.2020.05.022

[tpj15184-bib-0090] Yoshida, A. , Sasao, M. , Yasuno, N. , Takagi, K. , Daimon, Y. , Chen, R. ***et al***. (2013) TAWAWA1, a regulator of rice inflorescence architecture, functions through the suppression of meristem phase transition. Proceedings of the National Academy of Sciences of the United States of America, 110, 767–772. 23267064 10.1073/pnas.1216151110 PMC3545824

[tpj15184-bib-0091] Zhang, G. , Cowled, C. , Shi, Z. , Huang, Z. , Bishop‐Lilly, K.A. ***et al***. (2013) Comparative analysis of bat genomes provides insight into the evolution of flight and immunity. Science, 339, 456–460. 23258410 10.1126/science.1230835 PMC8782153

[tpj15184-bib-0092] Zhang, G. , Zhao, F. , Chen, L. , Pan, Y. , Sun, L. , Bao, N. ***et al***. (2019) Jasmonate‐mediated wound signalling promotes plant regeneration. Nature Plant, 5, 491–497. 10.1038/s41477-019-0408-x 31011153

[tpj15184-bib-0093] Zhao, Z. , Andersen, S.U. , Ljung, K. , Dolezal, K. , Miotk, A. , Schultheiss, S.J. ***et al***. (2010) Hormonal control of the shoot stem‐cell niche. Nature, 465, 1089–1092. 20577215 10.1038/nature09126

[tpj15184-bib-0094] Zhou, W. , Lozano‐Torres, J.L. , Blilou, I. , Zhang, X. , Zhai, Q. , Smant, G. ***et al***. (2019) A jasmonate signaling network activates root stem cells and promotes regeneration. Cell, 177, 942–956. 30955889 10.1016/j.cell.2019.03.006

[tpj15184-bib-0095] Zürcher, E. , Liu, J. , di Donato, M. , Geisler, M. & Müller, B. (2016) Plant development regulated by cytokinin sinks. Science, 353, 1027–1030. 27701112 10.1126/science.aaf7254

